# Clinical outcomes of extensive-stage small cell lung cancer patients treated with thoracic radiotherapy at different times and fractionations

**DOI:** 10.1186/s13014-021-01773-x

**Published:** 2021-03-04

**Authors:** Jinmin Han, Chengrui Fu, Baosheng Li

**Affiliations:** 1 Shandong First Medical University and Shandong Academy of Medical Sciences, Jinan, 250117 Shandong China; 2 Department of Radiation Oncology, Shandong Cancer Hospital and Institute, Shandong First Medical University and Shandong Academy of Medical Sciences, Jinan, 250117 Shandong China; 3 Department of Radiation Oncology, Tianjin Medical University Cancer Institute and Hospital, National Clinical Research Center for Cancer, Key Laboratory of Cancer Prevention and Therapy, Tianjin’s Clinical Research Center for Cancer, Tianjin, 300060 China

**Keywords:** Extensive small cell lung cancer, Thoracic radiation therapy, Radiation time, Radiation dose/fractionation, Prognosis

## Abstract

**Objective:**

The purpose of this study was to assess whether thoracic radiotherapy (TRT) combined with chemotherapy (CHT) showed promising anti-tumour activity in extensive-stage small cell lung cancer (ES-SCLC), to explore practice patterns for the radiation time and dose/fractionation and to identify prognostic factors for patients who would benefit from CHT/TRT.

**Methods:**

A total of 492 ES-SCLC patients were included from January 2010 to March 2019, 244 of whom received CHT/TRT. Propensity score matching was performed to minimize bias between the CHT/TRT and CHT-alone groups. Patients in the CHT/TRT group were categorized into four subgroups based on the number of induction CHT cycles. For effective dose fractionation calculations, we introduced the time-adjusted biological effective dose (tBED). Categorical variables were analysed with chi-square tests and Fisher’s exact tests. Kaplan–Meier curves were generated to estimate survival rates using the R-project. Multivariate prognostic analysis was performed with Cox proportional hazards models.

**Results:**

Patients who received CHT/TRT experienced improved overall survival (OS) (18.1 vs 10.8 months), progression-free survival (PFS) (9.3 vs 6.0 months) and local recurrence-free survival (LRFS) (12.0 vs 6.6 months) before matching, with similar results after matching. In the CHT/TRT group, the median LRFS times for the groups based on the radiation time were 12.7, 12.0, 12.0, and 9.0 months, respectively. Early TRT had a tendency to prolong PFS (median 10.6 vs 9.8 vs 9.0 vs 7.7 months, respectively, *p* = 0.091) but not OS (median 17.6 vs 19.5 vs 17.2 vs 19.0 months, respectively, *p* = 0.622). Notably, patients who received TRT within 6 cycles of CHT experienced prolonged LRFS (*p* = 0.001). Regarding the radiation dose, patients in the high-dose group (tBED > 50 Gy) who achieved complete response and partial response (CR and PR) to systemic therapy had relatively short OS (median 27.1 vs 22.7, *p* = 0.026) and PFS (median 11.4 vs 11.2, *p* = 0.032), but the abovementioned results were not obtained after the exclusion of patients who received hyperfractionated radiotherapy (all *p* > 0.05).

**Conclusion:**

CHT/TRT could improve survival for ES-SCLC patients. TRT performed within 6 cycles of CHT and hyperfractionated radiotherapy (45 Gy in 30 fractions) may be a feasible treatment scheme for ES-SCLC patients.

**Supplementary Information:**

The online version contains supplementary material available at 10.1186/s13014-021-01773-x.

## Background

Small cell lung cancer (SCLC) accounts for approximately 13–15% of primary lung cancers and is characterized by its highly aggressive nature, early dissemination and good response to treatment, with almost two-thirds of SCLC patients presenting in an extensive stage (ES) at the first clinical diagnosis [1, 2]. Four to six cycles of platinum-based chemotherapy (CHT) alone is the historic standard treatment for ES-SCLC, with thoracic radiation (TRT) and prophylactic cranial irradiation (PCI) considered for patients who achieved response despite controversy [3, 4]. Recently, the FDA approved immunotherapy (IO) as a front-line treatment option in combination with CHT given the results of the IMpower133 and CASPIAN trials [5, 6]. Moreover, the use of PCI may further decrease if IO can reduce the incidence of brain metastasis in ES-SCLC patients [7].

Prior studies have demonstrated that TRT plays a vital role in terms of regional control and improved survival for ES-SCLC patients. A previous study published by Jeremic et al. was the first to point out the importance of TRT in ES-SCLC but with less attention [8]. The CREST trial, despite the primary endpoint at 1 year not being met, illustrated a 10% 2-year improvement for patients who responded to CHT with subsequent TRT [9]. Subgroup analysis of the CREST trial concluded that TRT should not be offered to patients who achieved complete intrathoracic response [10]. In other separate secondary analyses, survival was improved in patients with 2 or fewer metastases, and the presence of liver and/or bone metastases was an important factor in identifying beneficiaries [11, 12]. Additionally, the RTOG 0937 study, which delayed progression but regrettably failed to improve 1-year overall survival (OS), reported no difference among patients who underwent TRT early or late [13]. Several retrospective analyses also suggested that TRT in combination with CHT was associated with long-term survival [14–18]. This treatment strategy was advocated for certain ES-SCLC patients both in the 2020 NCCN guidelines [19] and in the ASTRO 2020 guidelines [20]. Nevertheless, there is no clear consensus on the application of TRT for ES-SCLC to date. Especially in the absence of TRT, as a first-line treatment, IO with the incorporation of atezolizumab or durvalumab into the CHT scheme has prolonged survival, making the role of TRT even more unclear. Hence, we conducted this retrospective real-world study. The aims of this study were as follows: first, to characterize whether TRT added to CHT (CHT/TRT) showed promising anti-tumour activity in ES-SCLC; second, to explore the appropriate TRT time and optimal radiation dose/fraction for survival; and third, to identify prognostic factors influencing the clinical outcome for ES-SCLC patients to distinguish who would benefit from CHT/TRT.

## Materials and methods

### Patients and study design

We retrospectively registered ES-SCLC patients who were treated in Shandong Cancer Hospital between January 2010 and March 2019. Clinical information, including demographic details, Eastern Cooperative Oncology Group (ECOG) performance status (PS) score, metastatic sites, treatment information, and haematological and nonhaematological toxicities, was collected from electronic medical records. Eligible patients had to satisfy the following criteria: (1) histologically or cytologically confirmed as having SCLC and in the ES by imaging at the initial diagnosis; (2) at least two cycles of CHT regardless of TRT receipt; and (3) an ECOG PS score of 0–2. The exclusion criteria were as follows: (1) patients who received salvage radiotherapy due to recurrence; (2) a history of malignancy in other sites that affect survival; and (3) incomplete clinical data or lost to follow-up. Our study was approved by the Ethics Review Committee of Shandong Cancer Hospital.

### Treatment strategy

The CHT regimens were mainly platinum combined with etoposide. All patients were administered either 3D conformal radiotherapy (3D-CRT) or intensity-modulated radiation therapy (IMRT). The gross tumour volume (GTV) encompassed the primary tumour and the positive lymph nodes. The clinical target volume (CTV) was defined as the GTV with a 5 mm margin, and the planning target volume (PTV) was expanded from the CTV with a 5–8 mm margin. If the tumour lesion was too large to carry out a tolerable radiotherapy plan, a 5–10 mm margin was directly expanded on the basis of the GTV to form the planning gross target volume (PGTV). Considering different radiation fractionations and time efficiencies, we employed the time-adjusted biological effective dose (tBED) formula [21]: tBED = (nd) {1 + [d/(α/β)]} − [0.693t/(αTpot)], where n is the number of fractions, d represents the dose per fraction, α/β = 10, α = 0.3 Gy, t is the number of radiotherapy days, and Tpot is the potential doubling time (5.6 days) [22, 23].

### Assessment of the response and toxicity

Imaging examinations were required almost every 2 cycles of CHT, before or after TRT, or as clinical manifestations worsened. The tumour response to first-line treatment was assessed by the Response Evaluation Criteria in Solid Tumors (RECIST) version 1.1. Efficacy was classified as complete response (CR), partial response (PR), stable disease (SD), or progressive disease (PD) [24]. Toxic effects were assessed according to the Common Terminology Criteria for Adverse Events (version 4.0) [25].

### Statistical analysis

Statistical analysis was performed via SPSS version 24.0 software (IBM Corp). Propensity score matching (PSM) (1:1) was performed to ensure well-balanced characteristics between the CHT/TRT and CHT-alone groups. The propensity score was calculated by a multivariable logistic regression model, with TRT as the dependent variable and age, sex, ECOG PS score, smoking index, metastatic organs, number of metastases, brain metastasis, liver metastasis, bone metastasis, weight loss, and PCI as the covariates. Chi-square and Fisher’s exact tests were employed to compare baseline characteristics between groups. Survival information, including OS, progression-free survival (PFS) and local recurrence-free survival (LRFS), was collected until October 31, 2019. OS was calculated from the date of diagnosis to death or the period up to the observation point. PFS was defined as the time of diagnosis until disease progression or death. LRFS was defined as the date of diagnosis until the time of local recurrence or death. Kaplan–Meier curves including the numbers at risk were plotted using R-project (version 4.0.3, http://www.Rproject.org). Univariable and multivariate Cox regression analyses were performed to identify the potential predictors of ES-SCLC patients. All statistical analyses were two-sided, and a *P* value < 0.05 was considered statistically significant.

## Results

### Patient characteristics

After rigorous reviews, 492 patients met the eligibility criteria for the final analysis, 244 of whom received CHT/TRT and 248 received CHT alone. The clinical characteristics of the study cohorts were comparable after PSM (Additional file 1: Table S1).

The median follow-up duration of the CHT/TRT group was 33 months. In total, 196 patients received conventional fractionated radiotherapy (40–66 Gy at 1.8–2 Gy/fraction daily), 40 patients received hyperfractionated radiotherapy (45 Gy at 1.5 Gy/fraction twice per day), and 8 patients received hypofractionated radiotherapy (30–51 Gy at 3 Gy/fraction daily). PCI was given as 25 Gy or 30 Gy in ten fractions. A total of 98 patients had bone metastasis, 31 of whom accepted bisphosphonates and 28 of whom received palliative radiotherapy to relieve pain. A total of 148 patients had brain metastases, with more than 80% (121 patients) undergoing either whole-brain radiotherapy (WBRT) or stereotactic radiotherapy (SRT). Notably, 33 patients received IO or targeted therapy after recurrence.

Patients were apportioned to four groups regarding the number of induction CHT cycles prior to TRT. Group A received TRT before or at the second cycle of CHT (≤ 2 cycles, n = 41); group B received TRT from the third cycle to the fourth cycle of CHT (3–4 cycles, n = 78); group C received TRT from the fifth cycle to the sixth cycle of CHT (5–6 cycles, n = 92); and group D received TRT after the sixth cycle of CHT (> 6 cycles, n = 33). There were no differences in the distribution of most variables other than bone metastasis among the four groups. To determine whether escalated doses to TRT had any significant impact on the outcomes, patients were classified into low-dose and high-dose groups according to two previous studies [26, 27]. Patient characteristics are presented in Tables 1 and 2**.**

**Table 1:** Clinical characteristics of ES-SCLC patients based on the radiation time

Variables		≤ 2cycles	3-4cycles	5-6cycles	> 6 cycles	*p* value
Age, y	< 60	20	36	46	15	
	≥ 60	21	42	46	18	0.950
Sex	Male	34	60	68	30	
	Female	7	18	24	3	0.189
ECOG PS score	0–1	38	71	85	32	
	2	3	7	7	1	0.487
Smoking index	≥ 400	20	47	38	15	
	< 400	21	31	54	18	0.098
Metastatic organs	single	16	36	33	9	
	Multiple	25	42	59	24	0.266
Number of metastases	≤ 2	11	18	16	4	
	> 2	30	60	76	29	0.345
Brain metastasis	yes	25	46	55	22	
	no	16	32	37	11	0.891
Liver metastasis	yes	9	21	35	7	
	no	32	57	57	26	0.128
Bone metastasis	yes	18	22	38	20	
	no	23	56	54	13	0.014
Weight loss	yes	4	8	14	5	
	no	37	70	78	28	0.695
PCI	yes	3	6	5	2	
	no	38	72	87	31	0.641
Radiation dose	≤ 50 Gy	27	50	60	22	
	> 50 Gy	14	28	32	11	0.994

ES-SCLC, Extensive-stage small-cell lung cancer; ECOG PS, Eastern Cooperative Oncology Group performance status; PCI, Prophylactic cranial irradiation;

**Table 2:** Clinical characteristics of ES-SCLC patients based on the radiation dose

Variables		Low -dose	High-dose	*p* value
Age, y	< 60	77	40	
	≥ 60	82	45	0.838
Sex	Male	128	64	
	Female	31	21	0.344
ECOG PS score	0–1	149	77	
	2	10	8	0.374
Smoking index	≥ 400	79	41	
	< 400	80	44	0.829
Metastatic organs	Single	56	38	
	Multiple	103	47	0.147
Number of metastases	≤ 2	31	18	
	> 2	128	67	0.755
Brain metastasis	Yes	97	51	
	No	62	34	0.878
Liver metastasis	Yes	50	22	
	No	109	63	0.364
Bone metastasis	Yes	68	30	
	No	91	55	0.257
Weight loss	Yes	24	7	
	No	135	78	0.125
PCI	Yes	12	4	
	No	147	81	0.588

ES-SCLC, Extensive-stage small-cell lung cancer; ECOG PS, Eastern Cooperative Oncology Group performance status; PCI, Prophylactic cranial irradiation;

### Survival outcomes

**Fig. 1:**
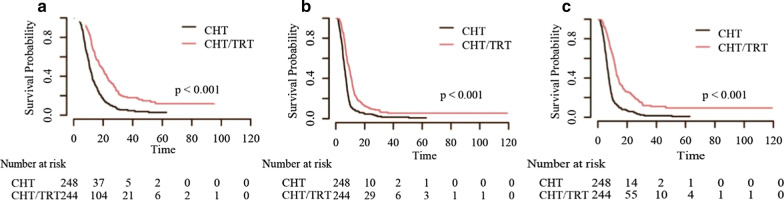
Kaplan–Meier survival curves of all patients in the CHT/TRT and CHT-alone groups before matching

**Fig. 2:**
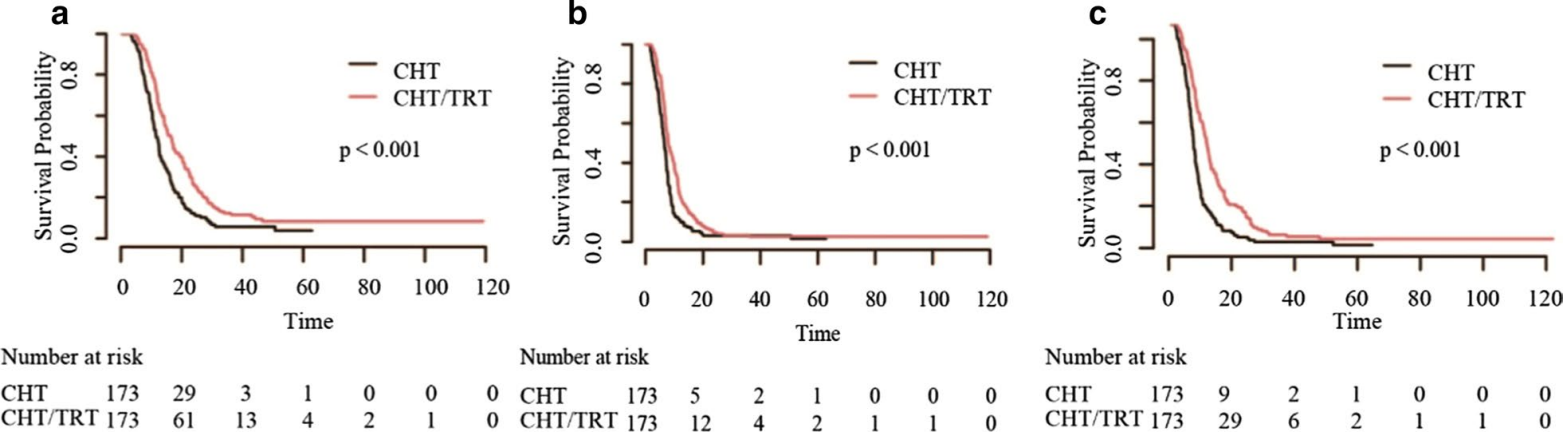
Kaplan–Meier survival curves of all patients in the CHT/TRT and CHT-alone groups after matching

**Fig. 3:**
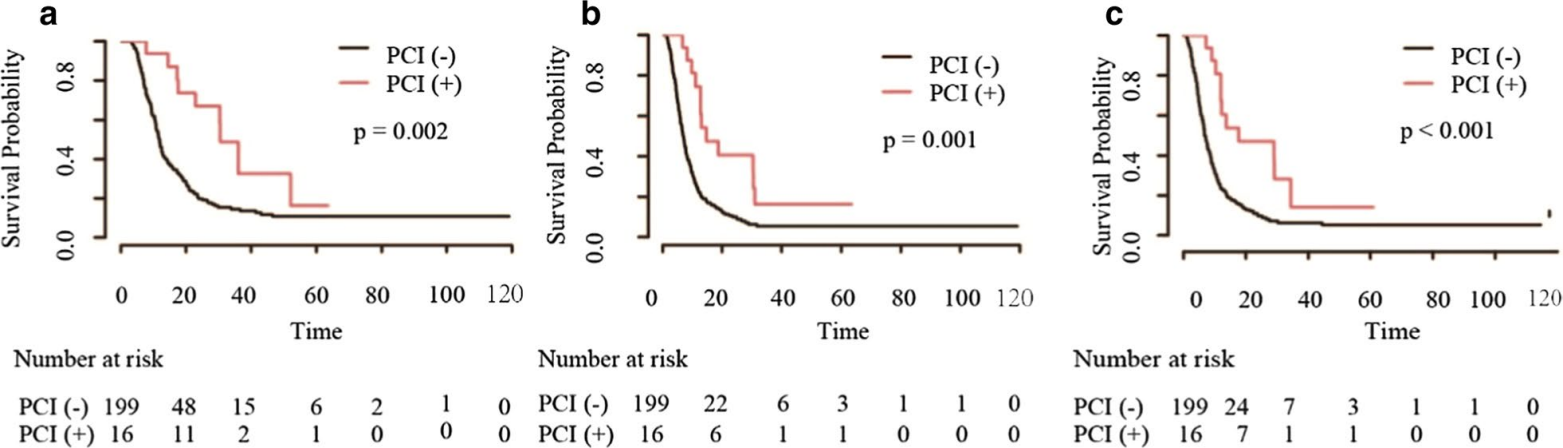
Kaplan–Meier survival curves of patients without brain metastasis who received PCI ( +) or did not receive PCI (−)

**Fig. 4:**
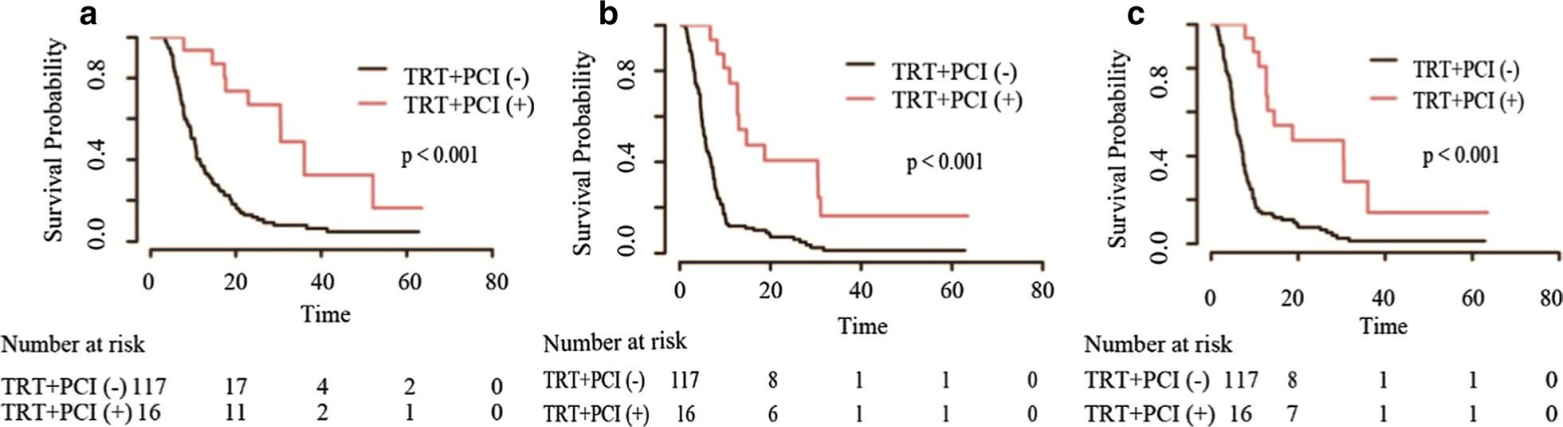
Kaplan–Meier survival curves of patients without brain metastasis who received TRT + PCI ( +) or did not receive TRT + PCI (−)

Patients who received CHT/TRT experienced longer OS (18.1 vs 10.8 months), PFS (9.3 vs 6.0 months) and LRFS (12.0 vs 6.6 months) than those who received CHT alone before matching (all *p* < 0.001, Fig. 1). The survival benefit remained significant for OS (16.4 vs 11.6 months), PFS (7.9 vs 6.5 months) and LRFS (10.6 vs 7.1 months) after matching (all *p* < 0.001, Fig. 2). In the subgroup analysis of patients without brain metastasis, a significant increase was observed in patients who received PCI compared with those who received non-PCI; the same was observed for patients who received TRT + PCI compared with those who received non-TRT + PCI (all *p* < 0.001, Figs. 3 and 4).

**Fig. 5:**
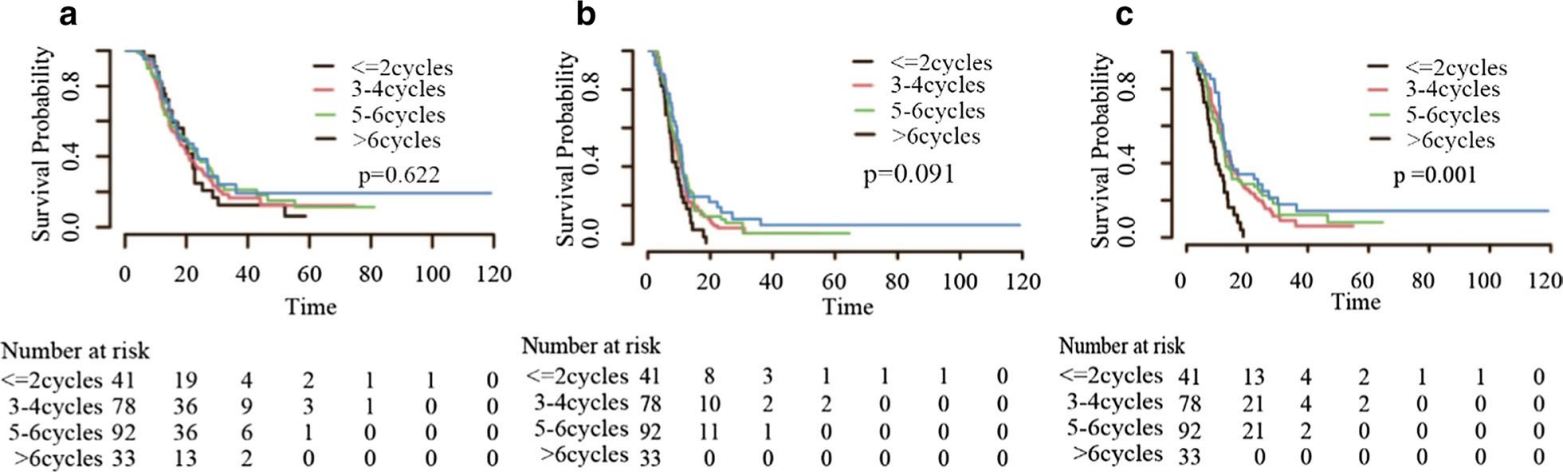
Kaplan–Meier survival curves of ES-SCLC patients based on the radiation time

**Fig. 6:**
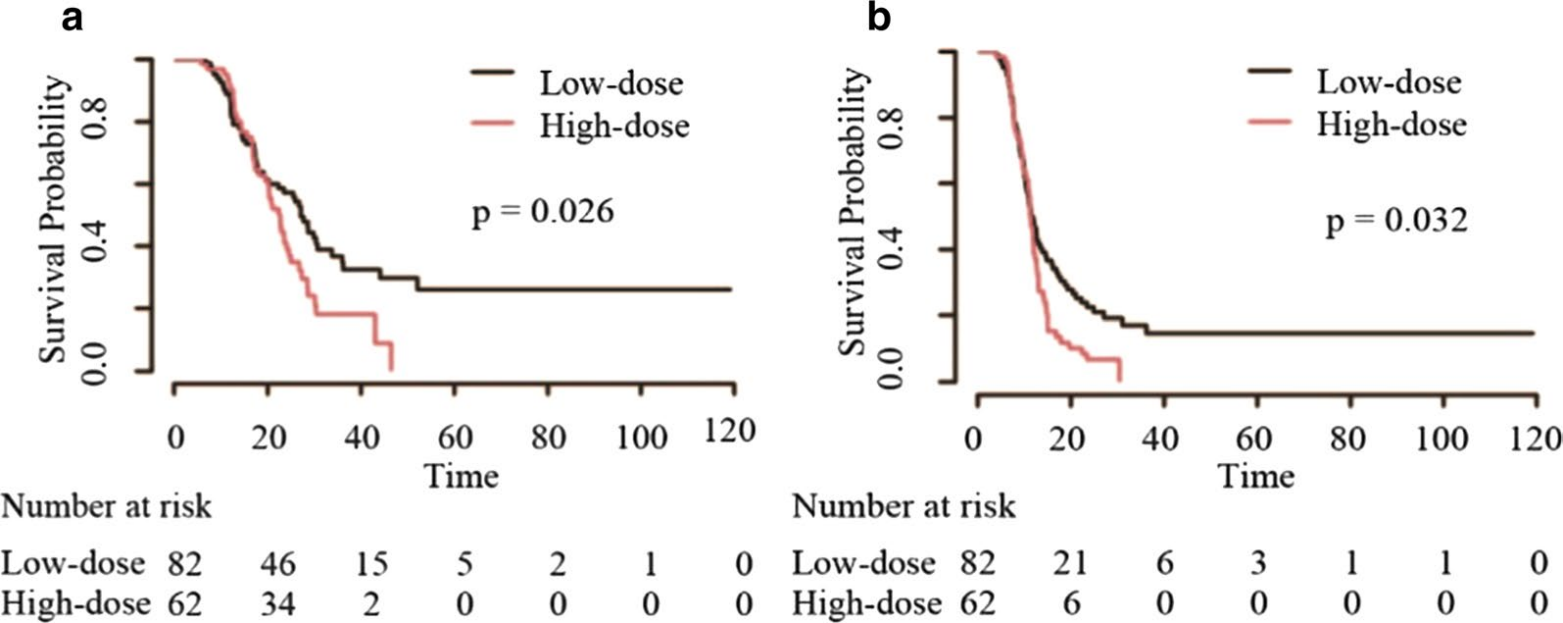
Kaplan–Meier survival curves of ES-SCLC patients who achieved CR and PR based on the radiation dose

**Fig. 7:**
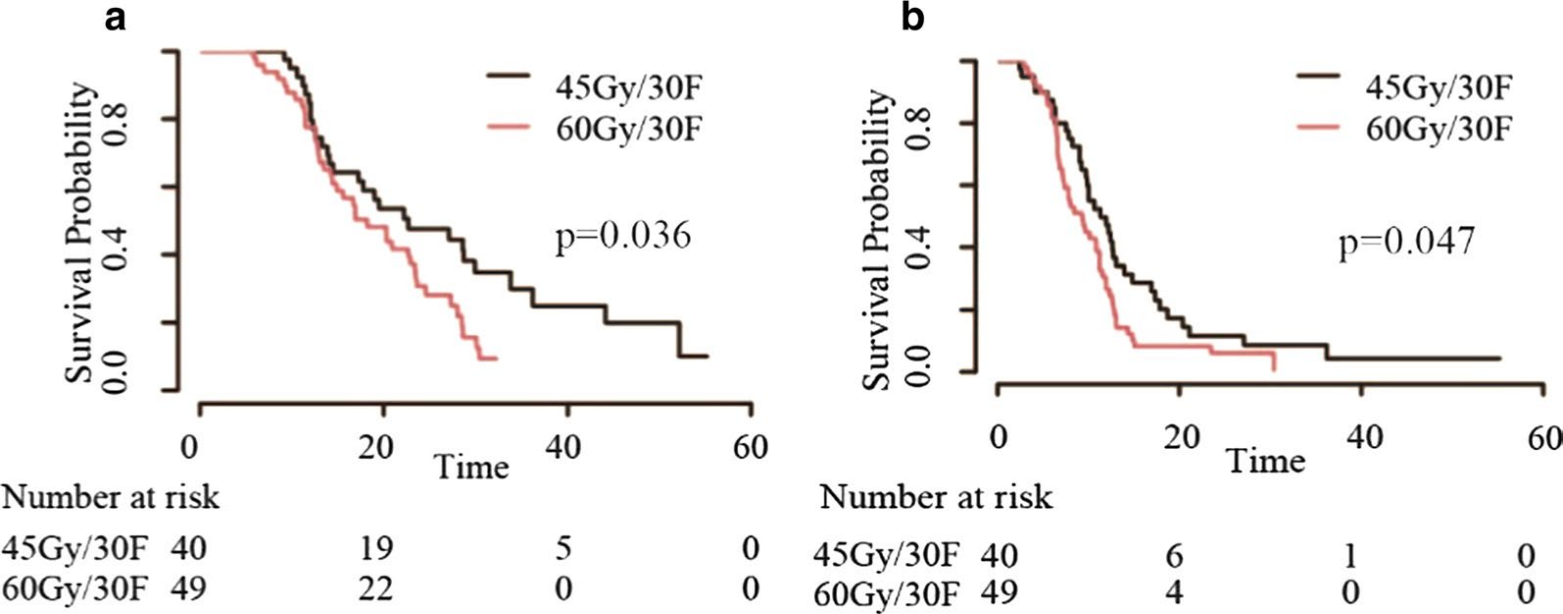
Kaplan–Meier survival curves of ES-SCLC patients who received 45 Gy/30 fraction or 60 Gy/30 fraction

We then attempted to explore the appropriate TRT time and optimal dose/fraction in the population who received CHT/TRT. With regard to the radiation time, the median LRFS (mLRFS) times based on the radiation time were 12.7, 12.0, 12.0, and 9.0 months, respectively. The median PFS (mPFS) times were 10.6, 9.8, 9.0, and 7.7 months, respectively. The median OS (mOS) times were 17.6, 19.5, 17.2, and 19.0 months, respectively. Patients who received TRT within 6 cycles of CHT had a better mLRFS than those who received TRT after 6 cycles of CHT (*p* = 0.001). Kaplan–Meier survival curves concerning the radiation time are shown in Fig. 5. Regarding the radiation dose, patients in the high-dose group had better OS (median 20.8 vs 17.3, *p* = 0.747), PFS (median 9.9 vs 9.0, *p* = 0.679) and LRFS (median 12.7 vs 11.4, *p* = 0.977), but the differences were not statistically significant (all *p* > 0.05). We further analysed the patients who achieved CR and PR to systemic therapy, with the low-dose group having better OS (median 27.1 vs 22.7, *p* = 0.026) and PFS (median 11.4 vs 11.2, *p* = 0.032) (Fig. 6). Unfortunately, the differences were not significant when patients who received hyperfractionated radiotherapy were excluded (all *p* > 0.05). Specifically, patients who received 45 Gy at 1.5 Gy/fraction twice per day experienced better OS (median 22.7 vs 18.2, *p* = 0.036) and PFS (median 11.3 vs 9.3, *p* = 0.047) than those who received 60 Gy radiotherapy at 2 Gy/fraction daily (Fig. 7). In addition, patients in the hypofractionated radiotherapy group had similar outcomes to those in the conventional fractionated radiotherapy group (all *p* > 0.05).

### Response to treatment and treatment failure

The ORRs according to the radiation time and radiation dose were 68.3% vs 60.3% vs 57.6% vs 48.5% (*p* = 0.377) and 72.9% vs 51.6% (*p* = 0.001), respectively. Regarding failure patterns, in the CHT/TRT group, 29 (11.9%) patients had progression in the thoracic area, 74 (30.3%) patients had progression at distant sites, and 90 (36.9%) patients developed regional and distant recurrence, whereas in the CHT-alone group, 35 (14.1%) patients had progression in the thoracic area, 25 (10.1%) patients had progression at distant sites, and 151 (60.9%) patients developed regional and distant recurrence. The local relapse rate was significantly decreased with the receipt of TRT (48.8% vs 75.0%); however, the distant control rate was disappointing (67.2% vs 71.0%). More specifically, the recurrence rates of these four groups were 70.7%, 73.1%, 81.5%, and 97.0%, respectively (*p* < 0.001), while no significant difference was found between the high-dose group and the low-dose group (78.6% vs 80.0%, *p* = 0.726).

### Prognostic factors influencing survival

The following factors were identified as significant prognostic factors for OS in univariate analysis: ECOG PS score (*p* = 0.013), smoking index (*p* = 0.014), number of metastases (*p* = 0.006), metastatic organs (*p* < 0.001), liver metastasis (*p* < 0.001), bone metastasis (*p* = 0.011), weight loss(*p* = 0.021), and PCI (*p* = 0.018). Next, multivariate analysis revealed that an excellent PS score and PCI were independent, favourable prognostic factors for OS. Liver metastasis, weight loss and the smoking index were adverse factors affecting prognosis in ES-SCLC patients (all *p* < 0.05). Details are presented in Table 3.

**Table 3:** Univariate and multivariate survival analyses of the prognostic factors for OS in patients receiving TRT

Variables	Univariate	Multivariate	
	*p* value	HR(95%CI)	*p* value
Age, y (≥ 60 vs < 60)	0.191	–	–
Sex (Male vs Female)	0.073	–	–
ECOG PS score (≤ 1 vs > 1)	0.013	0.52 (0.31,0.88)	0.013
Smoking index (< 400 vs ≥ 400)	0.014	1.56 (1.16,2.11)	0.003
Number of metastases (≤ 2 vs > 2)	0.006	1.01 (0.61,1.68)	0.962
Metastatic organs (Single vs Multiple)	< 0.001	1.52 (0.97,2.36)	0.065
Brain metastasis (yes vs no)	0.299	–	–
Liver metastasis (yes vs no)	< 0.001	1.71 (1.22,2.41)	0.002
Bone metastasis (yes vs no)	0.011	1.13 (0.82,1.56)	0.455
Weight loss (yes vs no)	0.021	1.60 (1.04,2.46)	0.034
PCI (yes vs no)	0.018	0.48 (0.24,0.96)	0.039

ECOG PS, Eastern Cooperative Oncology Group performance status; PCI, Prophylactic cranial irradiation; OS, overall survival; TRT, thoracic radiotherapy; HR, hazard ratio; CI, confidence interval

### Safety profile

In this study, side effects of grade II and above (haematologic toxicity, gastrointestinal toxicity, acute radiation-induced pneumonitis, and oesophagitis) were defined as toxic. Leucopenia was more frequent than other toxicities, and no treatment-related deaths occurred. No significant difference was observed among these four groups. Nausea/vomiting and TRT-induced oesophagitis were more common in the high-dose group than in the low-dose group. Haematologic and nonhaematologic toxicities are summarized in Table 4.

**Table 4:** Adverse events summarized by the time and dose of TRT

Toxic Effect/Grade	≤ 2 cycles	3–4 cycles	5–6 cycles	> 6 cycles	*p* value	Low-dose	High-dose	*p* value
*Haematologic toxicity grade ≥ 2*								
Leucopenia	27	52	49	18	0.244	90	56	0.159
Anemia	9	10	7	6	0.111	20	12	0.734
Thrombocytopenia	7	12	14	5	0.994	24	14	0.778
*Nausea/vomiting*								
Grade 0–1	33	62	79	24		137	61	
> Grade 2	8	16	13	9	0.388	22	24	0.006
*TRT-induced Oesophagitis*								
Grade 0–1	37	71	83	29		148	72	
> Grade 2	4	7	9	4	0.729	11	13	0.036
*TRT-induced Pneumonitis*								
Grade 0–1	35	64	77	25		133	68	
> Grade 2	6	14	15	8	0.714	26	17	0.476

TRT, thoracic radiotherapy

## Discussion

In the present study, TRT added to CHT in ES-SCLC patients was associated with long-term survival both before and after matching. We found that the mOS for patients treated with CHT/TRT was 18.1 months, which is similar to a retrospective study that demonstrated a comparable mOS (17 months) [14]. Two recent randomized phase III trials confirmed survival advantages in ES-SCLC patients who received both IO and CHT. IMpower133 was the first trial to show improved survival in patients treated with atezolizumab combined with CHT (mOS 12.3 months vs 10.3 months). The CASPIAN study also showed an improvement in survival, which reported an mOS of 13.0 months in the durvalumab combined with CHT group and 10.3 months in the CHT-alone group. Based on the above two studies, atezolizumab or durvalumab combined with CHT has become the preferred recommended protocol for ES-SCLC. Notably, patients could have PCI, but TRT was not allowed in these two studies. However, survival data from both studies did not show superior survival with CHT/IO compared to CHT/TRT. Whether IO combined with TRT could improve survival remains to be further determined, and the role of TRT is more difficult to determine with the inclusion of IO; furthermore, the optimal time and radiation dose have not been uniformly characterized.

With respect to the radiation time, the Jeremic trial reported a survival advantage when TRT was given after three cycles of CHT [8], whereas a retrospective study by Luo et al. did not show a significant benefit between early and late TRT [28]. We evaluated the efficacy of introducing TRT at different time points. An improvement, albeit not statistically significant, in PFS was found with earlier TRT compared to delayed TRT, suggesting that earlier TRT could prolong PFS and thus improve OS, although this benefit was not durable. Additionally, TRT within 6 cycles presented a significant difference in LRFS; therefore, TRT has been administered to enhance locoregional control. Further evaluation is required to determine whether TRT could provide a clear survival benefit. Several reasons may account for this fact. First, ES-SCLC is a systemic disease, and early TRT may be more effective in improving local control than extrathoracic control. Second, the unbalanced prognostic factor of bone metastasis may have resulted in a statistical disconformity. Finally, the number of patients in each group was small, and treatment regimens as second-line CHT after recurrence were inhomogeneous.

Whether a higher TRT dose could give rise to a favourable prognosis is still an unresolved question. Two recently published studies suggested that patients in the high-dose group had longer OS than those in the low-dose group [26, 27]. We found that patients treated with higher doses had better mOS, mPFS and mLRFS, but the differences were not statistically significant. We next analysed the patients who achieved CR and PR to systemic therapy but reached discordant conclusions; that is, patients in the low-dose group had superiority over those in the high-dose group. The different radiation fractionations employed may have led to this inconsistent result. Unlike the abovementioned studies, patients who received hyperfractionated radiotherapy were included in our study. Moreover, receiving TRT at 45 Gy/30 fractions twice per day translated into a survival benefit in contrast with receiving radiotherapy at 60 Gy/30 fractions daily, which was consistent with the findings of Luan et al. [29]. Thus, TRT at 45 Gy/30 fractions twice daily appears to be a feasible treatment scheme for ES-SCLC patients. An interesting finding was that patients in the hypofractionated radiotherapy group had similar prognoses to patients in the conventional fractionated radiotherapy group and acceptable adverse effects, bringing great convenience for patients with weak physical conditions. However, the number of patients who received hypofractionated radiotherapy was small, and more homogeneous studies are needed to confirm the results.

However, we focused on the independent predictors in ES-SCLC patients who received TRT, including the ECOG PS score, PCI, smoking index, liver metastasis and bone metastasis. The ECOG PS score is traditionally used to predict the outcome of SCLC patients, with two previous studies reporting a relatively short OS duration in patients with a poor PS score [30, 31]. Our results were in conformity with their findings and indicate that TRT confers a survival advantage in patients with a good PS score and that the treatment tolerance in patients with an excellent PS score could be better than that in those with a poor PS score; thus, it seems reasonable to select ES-SCLC patients with an excellent PS score for systemic therapy with TRT. PCI was also proven to be a prognostic factor for improved survival. Furthermore, OS was improved with TRT + PCI compared to non-TRT + PCI. However, PCI was administered to only 16 patients, making statistical comparisons difficult. Taken together, these results show that the relationship between PCI and survival needs to be further verified.

Concerning distant metastasis, two previous studies by Nakazawa K et al. and Ren Y et al. revealed that a single metastasis was associated with better OS than multiple metastases [32, 33]. In contrast, metastatic sites (multiple vs single) and the number of metastases in our analysis were significantly obvious in univariate analysis, but they did not affect survival in multivariate analysis. One possible reason was the different sample sizes of the two groups. Cai et al. and Qin et al. reported that patients diagnosed with liver metastasis had a significantly increased risk of death, while no benefit was found in patients without brain metastasis and bone metastasis [34, 35]. Our results were consistent with these results and confirmed that patients without liver metastasis had better OS than those with liver metastasis. A high proportion of patients with brain metastasis who underwent either WBRT or SRT had a prognosis similar to that of those without brain metastasis. Owing to timely therapy with diphosphonates and palliative radiotherapy, there was no significant difference in OS between patients with and without bone metastasis. Further studies are needed to formulate the therapeutic schedule of ES-SCLC patients with liver metastasis.

Needless to say, the smoking index was identified as a negative predictor of OS [36, 37]. Weight loss was considered the diagnostic criterion for cancer cachexia according to a previous study by Fearon et al. [38]. We speculate that weight loss in ES-SCLC patients may be associated with a heavy tumour burden, tumour progression or low food intake caused by chest pain and dyspnoea, leading to decreased quality of life and an increase in mortality. Furthermore, our study confirmed that CHT/TRT was well tolerated in patients with ES-SCLC.

As previously reported, integrating IO and TRT may potentiate a synergistic effect and possibly augment the anti-tumour immune response, resulting in locoregional control and enhancing the IO effect on extrathoracic metastasis [39–41]. One prospective study by Welsh JW et al. corroborated that pembrolizumab added to TRT was safe and well tolerated in ES-SCLC patients, with the risk of treatment-related complications being manageable [42]. It is necessary to conduct large-scale prospective cohort studies to put this treatment paradigm into practice for ES-SCLC.

In addition to the retrospective nature of our research, several other limitations should be acknowledged. First, the small number of patients in the subgroups limited the statistical analyses. Second, the radiation dose/fraction, diversified therapeutic modality after disease progression and radiation target volume schemes may have contributed to study bias. Third, no biomarker analysis was performed, and patients who were lost to follow-up were not included in the study. Further studies are warranted to clarify the findings of this study.

## Conclusion

Considering the current and previous reports, there is no doubt that TRT could improve survival in ES-SCLC patients. TRT performed within 6 cycles of CHT and delivered hyperfractionated (45 Gy in 30 fractions) may be an appropriate treatment scheme. Consolidation TRT could be an option for patients who undergo PCI, with no liver metastasis, with a satisfactory ECOG PS score, with no weight loss as well as those who cease smoking. Nevertheless, whether TRT and PCI are superior in the era of IO is unknown. Future prospective studies that establish adjunct immune checkpoint inhibitors are required to confirm this hypothesis.

## Supplementary Information

**Additional file 1.** **Table S1** Clinical characteristics of ES-SCLC patients in the CHT/TRT and CHT-alone groups.

## Data Availability

The datasets used and analyzed during the current study are available from the corresponding author on reasonable request.

## Abbreviations

SCLC: Small-cell lung cancer
ES-SCLC: Extensive-stage small-cell lung cancer
EP/EC/EL: Etoposide + cisplatin/etoposide + carboplatin/etoposide + lobaplatin
ECOG PS: Eastern Cooperative Oncology Group performance status
OS: Overall survival
PFS: Progression-free survival
LRFS: Local recurrence-free survival
mOS: Median OS time
mLRFS: Median LRFS time
mPFS: Median PFS time
PSM: Propensity score matching
RECIST: Response evaluation criteria in solid tumors
CHT: Chemotherapy
TRT: Thoracic radiotherapy
CHT/TRT: TRT added to CHT
PCI: Prophylactic cranial irradiation
tBED: Time-adjusted biological effective dose
3D-CRT: 3D conformal radiotherapy
IMRT: Intensity-modulated radiation therapy
WBRT: Whole brain radiotherapy
SRT: Stereotactic radiotherapy
GTV: Gross tumour volume
CTV: Clinical target volume
PTV: Planning target volume
PGTV: Planning gross target volume
CREST: The chest radiotherapy extensive-stage small cell lung cancer trial
CR: Complete response
PR: Partial response
SD: Stable disease
PD: Progressive disease
IO: Immunotherapy
