# Supplementary material for: Clinical outcomes of extensive-stage small cell lung cancer patients treated with thoracic radiotherapy at different times and fractionations
Source: Radiat Oncol. 2021 Mar 4;16:47. doi: 10.1186/s13014-021-01773-x (PMC7934361; doi:10.1186/s13014-021-01773-x)
Supplement: Supplementary file 1 — Additional file 1. Table S1 Clinical characteristics of ES-SCLC patients in the CHT/TRT and CHT-alone groups. [file 13014_2021_1773_MOESM1_ESM.docx]

**Table S1** Clinical characteristics of ES-SCLC patients in the CHT/TRT and CHT-alone groups

| Variables |  | Before matching | | | After matching | | |
| --- | --- | --- | --- | --- | --- | --- | --- |
|  |  | CHT/TRT  (n=248) | CHT-alone  (n=244) | p-  value | CHT/TRT  (n=173) | CHT-alone  (n=173) | p-value |
| Age, y | <60 | 117 | 96 |  | 74 | 74 |  |
|  | ≥60 | 127 | 152 | 0.039 | 99 | 99 | 1.000 |
| Sex | Male | 192 | 205 |  | 138 | 137 |  |
|  | Female | 52 | 43 | 0.264 | 35 | 36 | 0.894 |
| ECOG PS score | 0-1 | 226 | 227 |  | 158 | 159 |  |
|  | 2 | 18 | 21 | 0.654 | 15 | 14 | 0.846 |
| Smoking index | ≥400 | 120 | 142 |  | 93 | 89 |  |
|  | <400 | 124 | 120 | 0.073 | 80 | 84 | 0.667 |
| Metastatic organs | single | 94 | 65 |  | 51 | 53 |  |
|  | Multiple | 150 | 183 | 0.003 | 122 | 120 | 0.815 |
| Number of metastases | ≤2 | 49 | 22 |  | 19 | 20 |  |
|  | >2 | 195 | 226 | <0.001 | 154 | 153 | 0.865 |
| Brain metastasis | yes | 148 | 130 |  | 105 | 105 |  |
|  | no | 96 | 118 | 0.065 | 68 | 68 | 1.000 |
| Liver metastasis | yes | 72 | 126 |  | 68 | 70 |  |
|  | no | 172 | 122 | <0.001 | 105 | 103 | 0.826 |
| Bone metastasis | yes | 98 | 112 |  | 74 | 71 |  |
|  | no | 146 | 136 | 0.263 | 99 | 102 | 0.744 |
| Weight loss | yes | 31 | 54 |  | 28 | 30 |  |
|  | no | 213 | 194 | 0.008 | 145 | 143 | 0.773 |
| PCI | yes | 16 | 1 |  | 1 | 1 |  |
|  | no | 228 | 247 | <0.001 | 172 | 172 | 1.000 |

**Abbreviations:** ES-SCLC: Extensive-stage small-cell lung cancer; ECOG PS: Eastern Cooperative Oncology Group performance status; PCI: Prophylactic cranial irradiation;

CHT: chemotherapy; TRT: thoracic radiotherapy; HR: hazard ratio; CI: confidence interval
